# Supplementary material for: Tissue-Specific Expression of the Terpene Synthase Family Genes in Rosa chinensis and Effect of Abiotic Stress Conditions
Source: Genes (Basel). 2022 Mar 20;13(3):547. doi: 10.3390/genes13030547 (PMC8950262; doi:10.3390/genes13030547)
Supplement: Supplementary file 1 [file genes-13-00547-s001.zip › genes-1632645-supplementary.pdf]

Supplementary Materials

Tissue-Specific Expression of the Terpene Synthase Family Genes in *Rosa chinensis* and Effect of Abiotic Stress Conditions

Yuhang Yan [1], Mouliang Li [1], Xiaoni Zhang [3], Weilong Kong [3], Mohammed Bendahmane [2], Manzhu Bao [1] and Xiaopeng Fu [1,*]

[1] Key Laboratory of Horticultural Plant Biology, Ministry of Education, College of Horticulture and Forestry Sciences, Huazhong Agricultural University, Wuhan 430070, China

[2] Laboratoire Reproduction et Development des Plantes, UMR INRAE-CNRS-Lyon1-ENSL, Ecole Normale Supérieure Lyon, 69364 Lyon, France

[3] Guangdong Laboratory for Lingnan Modern Agriculture, Genome Analysis Laboratory of the Ministry of Agriculture, Agricultural Genomics Institute at Shenzhen, Chinese Academy of Agricultural Sciences, Shenzhen, Guangdong 518120, China

* Correspondence to: Xiaopeng Fu, fuxiaopeng@mail.hzau.edu.cn; Telephone: 86-159-2625-8658; Fax: 86-027-8728-2010

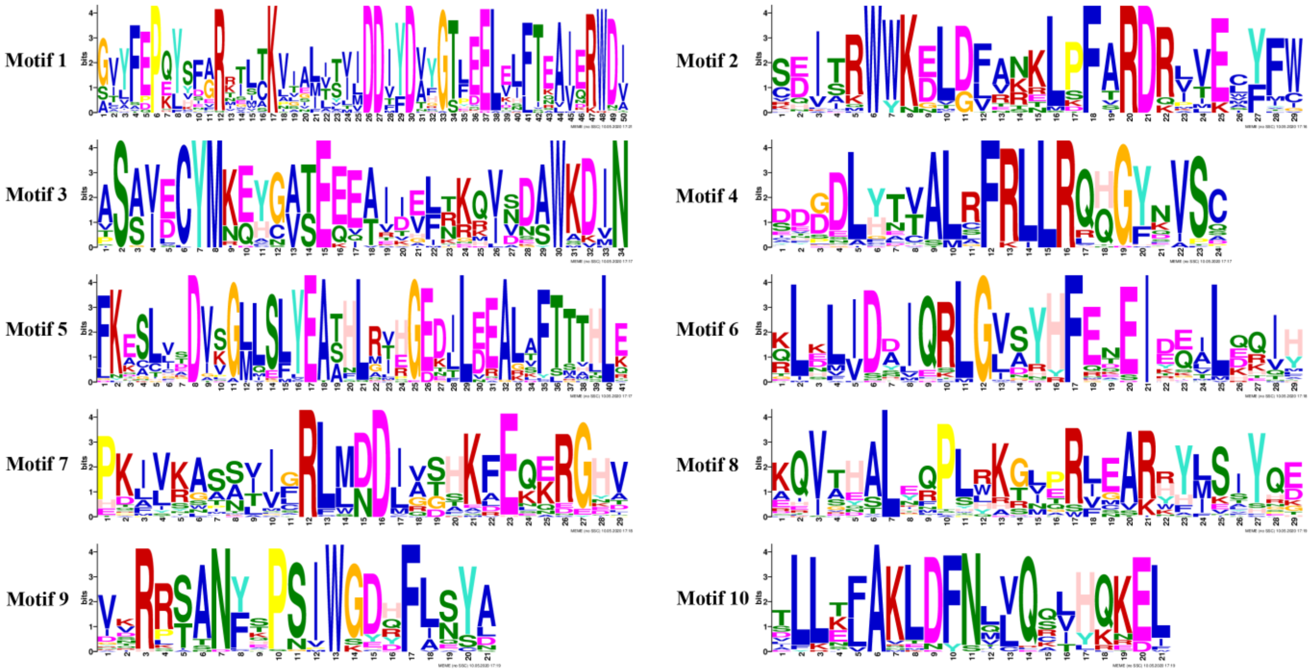

Figure S1. Ten conserved motifs in RcTPS proteins identified by MEME tool.

Table S1. Accession number of the TPS proteins represented in phylogenetic analysis.

Species	Gene ID	Accession number
Arabidopsis thaliana	*AtTPS01*	AT4G15870.1
	AtTPS02	AT4G16730.1
	AtTPS03	AT4G16740.1
	AtTPS04	AT1G61120.1
	AtTPS05	AT2G23230.1
	AtTPS06	AT1G70080.1
	AtTPS07	AT4G20200.1
	AtTPS08	AT4G20210.1
	AtTPS09	AT4G20230.1
	AtTPS10	AT2G24210.1
	AtTPS11	AT5G44630.1
	AtTPS12	AT4G13280.1
	AtTPS13	AT4G13300.1
	AtTPS14	AT1G61680.1
	AtTPS15	AT3G29190.1
	AtTPS16	AT3G29110.1
	AtTPS17	AT3G14490.1
	AtTPS18	AT3G14520.1
	AtTPS19	AT3G14540.1
	AtTPS20	AT5G48110.1
	AtTPS21	AT5G23960.1
	AtTPS22	AT1G33750.1
	AtTPS23	AT3G25830.1
	AtTPS24	AT3G25810.1
	AtTPS25	AT3G29410.1
	AtTPS26	AT1G66020.1
	AtTPS27	AT3G25820.1
	AtTPS28	AT1G48800.1
	AtTPS29	AT1G31950.1
	AtTPS30	AT3G32030.1
	AtTPS31	AT4G02780.1
	AtTPS32	AT1G79460.1
Solanum lycopersicum	*SlTPS03*	Solyc01g105870
	SlTPS04	Solyc01g105880
	SlTPS05	Solyc01g105890
	SlTPS07	Solyc01g105920
	SlTPS08	Solyc01g105960
	SlTPS09	Solyc06g059885
	SlTPS10	Solyc06g059910
	SlTPS12	Solyc06g059930
	SlTPS14	Solyc09g092470
	SlTPS16	Solyc07g008680

	SlTPS17	Solyc12g006570
	SlTPS18	Solyc08g005720
	SlTPS19	Solyc08g005670
	SlTPS20	Solyc08g005665
	SlTPS21	Solyc08g005640
	SlTPS24	Solyc07g066670
	SlTPS25	Solyc02g079890
	SlTPS27	Solyc02g079910
	SlTPS28	Solyc04g054380
	SlTPS31	Solyc01g101170
	SlTPS32	Solyc01g101180
	SlTPS33	Solyc01g101190
	SlTPS35	Solyc01g101210
	SlTPS36	Solyc06g060180
	SlTPS37	Solyc10g005410
	SlTPS38	Solyc02g079840
	SlTPS39	Solyc10g005390
	SlTPS40	Solyc06g084240
	SlTPS41	Solyc08g005710
	SlTPS46	Solyc03g006550
	SlTPS47	Solyc03g007730
	SlTPS48	Solyc04g051620
Malus domestica	*MdTPS01*	MDP0000120176
	MdTPS04	MDP0000130635
	MdTPS05	MDP0000161084
	MdTPS06	MDP0000203143
	MdTPS07	MDP0000205617
	MdTPS08	MDP0000225361
	MdTPS09	MDP0000233869
	MdTPS11	MDP0000248152
	MdTPS12	MDP0000265187
	MdTPS13	MDP0000274847
	MdTPS14	MDP0000274848
	MdTPS15	MDP0000276976
	MdTPS16	MDP0000295452
	MdTPS17	MDP0000317119
	MdTPS19	MDP0000670935
	MdTPS20	MDP0000702120
	MdTPS21	MDP0000746466
	MdTPS24	MDP0000154813
	MdTPS25	MDP0000199152
	MdTPS27	MDP0000322688
	MdTPS30	MDP0000147908
	MdTPS38	MDP0000321353

	MdTPS39	MDP0000192151
	MdTPS40	MDP0000192374
	MdTPS42	MDP0000227442
	MdTPS44	MDP0000318427
	MdTPS46	MDP0000828007
	MdTPS47	MDP0000245233
	MdTPS48	MDP0000267906
	MdTPS49	MDP0000293842
	MdTPS50	MDP0000298903
	MdTPS51	MDP0000308115
	MdTPS52	MDP0000398063
	MdTPS53	MDP0000561715
	MdTPS54	MDP0000562538

TPS proteins from *A. thaliana, S. lycopersicum* and *M. domestica* were obtained from their genome database. *A. thaliana*: https://www.arabidopsis.org/; *S. lycopersicum*: https://solgenomics.net/; *M. domestica*: https://www.rosaceae.org/.

Table S2. The FPKM values of *RcTPS* genes in different *R. chinensis* tissues.

Gene ID	Root	Stem	Leaf	Stamen	Pistil	FB_GP	FB_CP	FB_PP	OF_PP
RcTPS01	0.4802	0.1833	0.0000	2.7108	2.0556	0.1846	0.8767	0.4174	6.9958
RcTPS02	1.1422	1.6723	0.1068	0.3283	2.5626	0.2421	0.1205	0.1480	0.5041
RcTPS03	0.2620	0.2428	0.0000	0.0000	0.0000	0.0000	0.0000	0.0000	0.0000
RcTPS04	0.0000	0.0000	0.4840	0.0000	0.1796	0.0000	0.0000	0.0000	0.0000
RcTPS05	0.0000	0.5817	1.4969	0.0000	0.5264	0.0000	0.0083	0.0000	0.0000
RcTPS06	0.9880	0.0510	0.0348	547.1979	114.1788	0.6490	0.6222	12.6447	1221.0065
RcTPS07	0.0000	0.3177	1.8180	0.0262	0.0000	0.0000	0.0000	0.0000	0.0102
RcTPS08	0.0000	0.0000	0.0000	0.0218	0.3055	0.0000	0.0000	0.0000	0.0133
RcTPS09	0.0000	0.0000	0.0000	0.0000	0.0000	0.0000	0.0000	0.0000	0.9013
RcTPS10	0.0127	0.0000	0.0000	0.0000	0.0000	0.0000	0.0000	0.0000	0.0000
RcTPS11	0.0501	0.0195	0.0256	0.0000	0.2189	0.0000	0.0000	0.0000	0.0000
RcTPS12	0.0000	0.0000	0.0000	0.0000	0.0000	0.0138	0.0000	0.0000	0.0000
RcTPS13	0.0000	0.0000	0.3189	0.0000	0.3909	0.0000	0.1114	0.0451	0.0000
RcTPS14	0.0000	0.0000	0.0000	0.3365	0.1824	0.0000	0.1648	12.6921	10.6036
RcTPS15	0.0000	29.2513	0.8889	0.0000	11.9935	0.0141	0.0347	0.0000	0.0405
RcTPS16	0.0624	0.1774	0.0000	201.5982	14.2189	3.6696	5.2582	3632.6967	3881.7549
RcTPS17	0.0000	0.0000	0.0000	0.0000	0.0000	0.0000	0.0000	0.0000	0.0000
RcTPS18	0.0993	3.5836	35.7958	0.0366	0.2065	0.0000	0.0000	0.0000	0.1078
RcTPS19	0.0000	0.0182	0.0000	0.6362	0.8910	0.8415	7.4730	9.4191	0.5071
RcTPS20	0.0000	0.0000	0.0000	1.6356	9.9552	23.9863	199.1275	62.9024	0.4252
RcTPS21	0.0617	0.0000	2.8209	0.0220	2.4499	0.0000	0.0141	0.0000	0.0076
RcTPS22	0.0000	0.0338	1.0356	0.0000	1.1461	0.0201	0.0215	0.0000	0.0000
RcTPS23	0.0000	0.0000	0.0000	0.3350	0.0000	0.0000	0.0041	0.0067	0.0000
RcTPS24	0.0654	58.9367	71.0150	0.0000	0.1474	0.3892	0.0162	0.0000	0.0070
RcTPS25	7.2104	0.7112	0.0198	0.0354	0.2088	0.0000	0.0091	0.0000	0.0083
RcTPS26	0.0000	0.0000	0.0000	0.0000	0.0000	0.0000	0.0000	0.0000	0.0028
RcTPS27	0.0000	0.0000	0.8347	0.0000	0.9326	0.0000	0.0000	0.0000	0.0031
RcTPS28	0.0000	0.0845	0.8170	0.0000	0.0358	0.0108	0.0000	0.0169	0.2372
RcTPS29	0.0000	0.0000	0.0000	0.0000	0.0000	0.0000	0.0000	0.0000	0.0000
RcTPS30	0.8409	0.0000	0.0000	0.0000	0.3194	0.0000	0.0166	0.0000	0.0000
RcTPS31	7.7600	10.1484	3.3916	2.7991	19.2997	4.6147	3.1788	1.9191	12.5750
RcTPS32	0.0563	0.0000	0.0000	0.0000	0.0000	0.0000	0.0000	0.0000	0.0000
RcTPS33	0.0000	0.0000	0.0000	0.0000	0.3197	0.0000	0.0000	0.0000	0.0094
RcTPS34	0.0000	0.1045	0.0298	0.2680	0.0096	0.0171	0.0267	40.8715	67.3302
RcTPS35	0.1878	0.0804	0.6055	0.0908	0.8645	0.1448	0.0103	0.0128	1.8238
RcTPS36	0.3386	0.0182	0.0280	235.8967	27.4991	0.2153	0.2245	4.3817	581.9530
RcTPS37	0.1074	0.1879	10.0093	0.3588	5.2917	36.4463	56.8342	13.3891	0.2131

RcTPS38	0.0844	0.0000	0.0000	0.0000	0.0000	0.0000	0.0000	0.0000	0.0000
RcTPS39	0.0000	0.1056	0.0000	0.0086	0.0632	0.0000	0.0000	0.0000	0.0000
RcTPS40	0.0427	0.0232	0.2180	0.0000	1.2654	0.0104	0.0088	0.0000	0.4627
RcTPS41	0.0000	0.0000	0.0000	0.0279	0.2976	0.0000	0.0139	0.0445	0.1959
RcTPS42	0.0000	0.0000	0.1562	0.0000	0.0000	0.0000	0.0000	0.0000	0.0000
RcTPS43	0.5753	0.0130	0.0166	0.0000	0.1066	0.0000	0.0000	0.0000	0.0000
RcTPS44	0.0000	0.0000	0.0000	0.0242	0.0000	0.0000	0.0000	0.0085	30.7075
RcTPS45	0.0000	0.0000	0.0000	0.0000	0.0000	0.0000	0.0000	0.0000	0.0000
RcTPS46	0.0000	0.0000	0.0000	0.8824	0.0456	0.0000	0.1746	0.1187	45.8209
RcTPS47	0.0891	0.2064	0.1773	0.0039	0.0445	0.2056	0.1006	0.0000	0.0000
RcTPS48	0.2596	0.0793	0.1386	0.0000	0.0078	0.0000	0.0000	0.0000	0.0000
RcTPS49	0.0149	0.1469	1.1668	0.2792	0.5457	0.0627	0.0439	0.2053	0.2756

All FPKM values were downloaded from NCBI under BioProject PRJNA546486 and BioProject PRJNA351281. FB_GP: green petals in the flower buds; FB_CP: color-changing petals in the flower buds; FB_PP: pink petals in the flower buds; OF_PP: pink petals of the open flowers.

Table S3. The expression values of *RcTPS* genes under 200 mM mannitol treatment for 48 h.

Gene ID	CK	12 h	24 h	48 h
RcTPS01	20.5331±4.7816 a	8.7991±1.1336 c	12.6704±6.8054 bc	15.4782±5.4544 ab
RcTPS02	18.331±3.1179 b	23.5195±7.6909 b	58.9961±24.095 a	70.6734±20.1988 a
RcTPS03	12.8167±6.5792 b	17.2363±0.9203 b	40.4733±15.2319 a	53.3257±14.3186 a
RcTPS04	2.18±0.5864 b	2.4271±0.2411 b	6.0198±2.0727 a	5.8405±2.1489 a
RcTPS05	49.5417±3.4823 b	59.2808±1.1228 b	90.6046±36.8779 a	99.136±20.6175 a
RcTPS06	7996.0724±795.5345 a	1738.8111±229.9443 b	1603.6049±411.3078 b	639.2552±184.6102 c
RcTPS07	519.5228±101.4995 a	112.9524±33.4952 c	279.9107±164.275 b	171.9421±50.2511 bc
RcTPS08	7.7494±0.4599 b	8.0603±0.607 b	12.1067±6.0635 a	12.1015±2.0397 a
RcTPS09	15.9412±1.7627 b	12.9988±4.2955 b	27.5294±10.8596 a	34.8638±9.4434 a
RcTPS10	6.6234±0.6723 b	3.4541±4.2148 b	27.7012±15.4526 a	40.2797±15.1221 a
RcTPS11	38.6201±6.5629 b	21.2161±4.3192 b	169.0793±79.2691 a	39.1831±10.3998 b
RcTPS12	2.1176±0.3866 b	6.476±0.1047 b	60.1771±25.5669 a	22.131±6.1822 b
RcTPS13	3.1537±1.1771 b	1.6783±0.3763 b	18.1839±6.2777 a	3.8568±0.4757 b
RcTPS14	360.7312±60.0087 a	127.0249±9.2762 b	180.2859±67.3997 b	22.1345±2.2816 c
RcTPS15	10.9948±1.7483 b	6.4514±0.9512 b	58.0695±17.8909 a	17.1635±4.5193 b
RcTPS16	473.9112±42.0035 a	132.2631±1.3732 b	183.9881±85.3515 b	17.0735±6.4467 c
RcTPS17	22.1986±13.441 bc	16.111±0.8298 c	125.5594±38.7968 a	51.2007±14.5989 b
RcTPS18	4.1986±1.5662 b	1.1068±0.5374 b	20.7008±10.4711 a	8.1014±3.3301 b
RcTPS19	3.9527±1.3494 b	3.4858±0.3965 b	31.8489±17.3609 a	10.9224±2.683 b
RcTPS20	4.3357±0.2762 bc	1.9105±0.0489 c	23.6066±9.6961 a	10.8156±1.4063 b
RcTPS21	22.7627±1.6565 c	29.6214±0.8798 c	57.6954±32.7117 b	93.8743±28.7302 a
RcTPS22	9.033±0.7107 bc	5.1144±0.685 c	11.4952±5.5718 b	16.8309±4.4958 a
RcTPS23	49.5186±5.8605 bc	45.1601±7.2513 c	87.4396±44.0575 ab	114.8459±29.8097 a
RcTPS24	9.1626±0.0845 b	7.0602±2.5701 b	11.2967±4.7189 b	18.7653±5.0947 a
RcTPS25	7.5702±0.7226 b	6.9436±1.5336 b	11.5203±4.9061 b	18.9715±7.8756 a
RcTPS26	2.7738±0.746 a	2.7544±0.3358 a	4.621±2.871 a	6.4414±4.1579 a
RcTPS27	8.1708±0.9611 bc	7.074±1.4002 c	13.0486±2.5679 b	19.2522±6.1437 a
RcTPS28	2.4619±0.6741 b	2.9949±1.0024 b	4.5918±2.0553 b	7.4907±1.565 a
RcTPS29	16.1867±0.7108 a	6.7123±1.3893 c	11.3706±2.5894 b	16.235±4.1568 a
RcTPS30	19.5066±3.7325 b	7.2482±0.7249 b	36.6826±17.959 a	19.6799±2.2535 b
RcTPS31	8.545±1.5001 b	6.544±1.6609 b	16.6796±2.3667 a	13.6975±5.2567 a
RcTPS32	23.0706±13.4697 b	19.8271±4.3352 b	58.434±49.7603 a	42.9171±3.3638 ab
RcTPS33	17.8672±3.342 b	11.5845±0.9815 b	32.8225±14.4217 a	24.6713±4.9581 ab
RcTPS34	69.4014±1.1282 a	7.1451±0.1836 bc	13.6169±2.3271 b	6.9581±1.4451 c
RcTPS35	23.2471±1.5824 bc	16.4199±1.6223 c	62.6582±22.1877 a	34.97±7.7386 b
RcTPS36	7133.4921±694.4568 a	1351.788±189.3819 b	1300.8403±764.9858 b	601.2937±146.1674 c
RcTPS37	6.4494±1.0472 b	4.0177±0.246 b	12.0027±8.7822 a	10.1488±1.76 ab

RcTPS38	18.5272±5.0603 b	17.0176±0.6219 b	80.742±55.1955 a	48.0462±14.7383 ab
RcTPS39	3.1277±0.3027 b	1±1.0915 c	4.6004±1.5759 a	3.7673±0.9437 ab
RcTPS40	19.4502±1.7878 b	17.8552±2.215 b	60.3449±21.9262 a	45.6461±4.2377 a
RcTPS41	9.5682±1.3574 b	12.2559±6.0865 b	39.1778±21.8579 a	32.0873±9.8642 a
RcTPS42	16.2119±1.6863 b	12.0749±1.597 b	38.0824±15.5616 a	24.1334±5.8253 b
RcTPS43	42.2961±1.6986 a	35.6968±5.3958 a	85.3709±20.4801 a	87.3782±35.1079 a
RcTPS44	4877.0452±1026.0816 a	1300.904±121.7703 b	682.4764±240.143 b	335.5978±81.0104 b
RcTPS45	3.0769±0.2735 bc	1.9469±0.2777 c	5.0038±2.1492 a	4.3606±1.2688 ab
RcTPS46	38.3566±5.232 ab	20.6318±4.603 b	51.4189±24.0382 a	45.5292±13.2274 a
RcTPS47	3.3551±0.5456 b	4.7641±0.611 b	7.3229±2.2378 a	8.5795±1.5639 a
RcTPS48	21.6899±1.6394 b	22.3846±0.9558 b	48.4381±52.4429 a	44.9235±14.11 ab
RcTPS49	6.263±0.3611 b	5.5414±0.6164 b	14.6623±5.7012 a	11.141±3.0061 a

Means with different letters (a, b and c) are significantly different (P <0.05, one-way ANOVA along with Duncan's multiple range test).

Table S4. The expression values of *RcTPS* genes under heat treatment (35 °C) for 24 h.

Gene ID	CK	6 h	12 h	24 h
RcTPS01	11.5039±3.8756 b	29.8422±5.9904 b	66.2849±53.2353 a	15.8401±0.8598 b
RcTPS02	9.712±1.2102 c	52.842±4.6038 a	44.1836±12.3975 a	28.0483±4.3715 b
RcTPS03	1.6417±0.4766 c	8.7708±1.638 a	4.7201±1.0805 b	4.6555±0.1967 b
RcTPS04	0.5204±0.1104 b	1.1833±0.2696 a	1.5677±0.6244 a	0.6281±0.1449 b
RcTPS05	8.0182±0.7837 b	17.8516±1.1161 a	21.1097±3.995 a	9.4756±1.923 b
RcTPS06	6494.7551±1390.5278 a	2618.0218±1132.1302 b	7143.3291±1496.8593 a	1389.6367±453.9075 b
RcTPS07	3501.6264±215.9918 a	633.1079±169.5449 b	407.7718±121.7547 b	336.0514±63.2676 b
RcTPS08	1.657±0.4037 bc	2.6941±0.2347 ab	2.7826±1.5016 a	1.43±0.0761 c
RcTPS09	2.5663±0.3879 b	7.2374±0.828 a	7.0612±2.075 a	3.8416±0.3154 b
RcTPS10	2.8853±0.4956 b	6.5056±0.8094 a	4.9292±1.6261 a	3.4531±0.3983 b
RcTPS11	7.2842±0.3087 b	18.1794±2.7322 a	21.6548±4.1929 a	9.6496±0.8097 b
RcTPS12	99.0999±29.9337 a	20.3112±3.7643 b	28.7624±6.8542 b	10.7811±0.9188 b
RcTPS13	0.8642±0.0848 a	1.7811±0.6151 a	2.0438±1.3906 a	0.9454±0.1674 a
RcTPS14	2188.146±116.1929 a	988.619±63.4937 c	1741.9887±356.9524 b	524.7555±57.7652 d
RcTPS15	2.7021±0.0963 b	7.188±0.0358 a	6.8489±2.3941 a	3.8154±0.5331 b
RcTPS16	2697.7121±247.0582 a	1216.3161±118.0096 c	1983.6057±502.6737 b	645.6162±50.9965 d
RcTPS17	5.8643±0.8049 b	17.2712±3.4539 a	16.9445±4.4601 a	9.1675±0.7251 b
RcTPS18	11.937±1.3384 a	2.7618±0.4802 c	4.5795±0.9807 b	1.4659±0.2503 d
RcTPS19	1.4259±0.1323 b	2.678±0.2881 b	4.0061±1.4873 a	1.4215±0.0798 b
RcTPS20	1.1568±0.2836 b	2.419±0.1731 a	2.1793±0.6107 a	1.284±0.1068 b
RcTPS21	6.8669±0.9697 b	17.9767±3.2595 a	18.0276±4.3466 a	9.542±0.7855 b
RcTPS22	1.5266±0.1974 c	3.2291±0.1856 b	4.8003±0.9335 a	1.714±0.2378 c
RcTPS23	10.6461±1.4055 b	27.907±5.4184 a	30.623±3.1869 a	14.8129±0.882 b
RcTPS24	2.1177±0.2027 c	3.7679±1.1102 b	5.0037±0.6059 a	2±0.635 c
RcTPS25	1.8733±0.1646 c	4.1157±0.3973 b	5.7183±1.7967 a	2.1846±0.4395 c
RcTPS26	0.7775±0.0136 b	1.753±0.6441 a	1.6531±1.0835 a	0.9305±0.1957 b
RcTPS27	7.6328±0.267 a	4.3416±0.3846 b	5.0488±1.5388 b	2.3045±0.1411 c
RcTPS28	0.7445±0.0843 a	1.7357±0.8829 a	1.9262±1.2159 a	0.9213±0.3126 a
RcTPS29	1.556±0.1993 c	3.3962±0.4646 b	4.5196±1.2795 a	1.8027±0.2674 c
RcTPS30	1.6081±0.2174 c	3.4952±0.5572 b	4.4585±0.5265 a	1.8553±0.1287 c
RcTPS31	4.0043±0.7943 b	22.8142±5.2528 a	24.1365±7.8372 a	12.1097±1.0288 b
RcTPS32	6.9291±1.2982 b	12.8019±3.2325 a	13.2998±2.7336 a	6.7952±0.6712 b
RcTPS33	2.4609±0.5045 b	6.9668±1.6668 a	8.6212±2.216 a	3.698±0.5681 b
RcTPS34	901.4146±37.8652 a	49.2625±13.0259 c	355.8609±101.1625 b	26.1484±6.0341 c
RcTPS35	4.4192±0.2982 b	17.42±4.2061 a	22.9259±4.4542 a	9.2465±0.9397 b
RcTPS36	7306.6674±1601.0915 a	2917.4335±670.9878 b	7325.2897±1567.9929 a	1548.5633±178.9693 b
RcTPS37	1.3375±0.1051 b	2.8168±0.7825 a	3.8847±1.2246 a	1.4952±0.2956 b

RcTPS38	5.9847±1.2547 b	9.0894±1.2624 b	11.8641±6.6359 a	4.8246±0.5512 b
RcTPS39	0.6029±0.2086 c	1.1704±0.0996 b	1.9731±0.3613 a	0.6212±0.0804 c
RcTPS40	4.0866±1.1188 c	15.969±3.082 a	16.8241±1.861 a	8.4763±0.4397 b
RcTPS41	3.2502±0.5984 b	16.8037±15.18 a	16.8454±3.5986 a	8.9193±6.1001 ab
RcTPS42	14.4618±4.3306 b	25.9899±5.1967 b	210.9919±71.3898 a	13.7953±1.0957 b
RcTPS43	7.8003±0.1406 c	15.5598±2.7896 b	25.6649±6.1572 a	8.2591±1.0598 c
RcTPS44	6605.7785±952.8025 a	2539.8493±240.6271 b	5455.03±1778.2561 a	1348.1429±142.4022 b
RcTPS45	0.5493±0.1917 b	1±0.4249 b	1.237±0.3499 a	0.5308±0.1865 b
RcTPS46	15.2228±1.4787 a	10.4708±2.1117 b	12.4362±2.6809 ab	5.5579±1.4562 c
RcTPS47	0.8066±0.2339 b	1.4732±0.1963 b	2.4749±0.957 a	0.782±0.1034 b
RcTPS48	3.2886±0.3821 c	16.1051±2.3015 a	14.289±4.3418 a	8.5485±0.4374 b
RcTPS49	0.8599±0.2682 b	1.9482±2.1213 ab	3.3787±1.8338 a	1.0341±1.0085 b

Means with different letters (a, b and c) are significantly different (P <0.05, one-way ANOVA along with Duncan's multiple range test).

Table S5. Primers used for qRT-PCR analysis.

Gene ID	Forward primer (5' to 3')	Reverse primer (5' to 3')
RcTPS01	AGCCTCACTCATCCAACGCAATG	TTCCACCACCTAGCAATCTCACAC
RcTPS02	TCATTTGCCTTGGGACCGATTGTG	TCGAAACTCCGTACAGCCTCCTC
RcTPS03	AACCAACCGCCGTGCCAATATC	CTTCCCAACCCGTGTGAATCCATC
RcTPS04	CATTCAGCGCTTGGGGGTAT	AGAGCAACCGTGTGAAGGTC
RcTPS05	TACCACCACTCATCTCCAGACTGC	CATGTAACGCCGAGCACCTAACC
RcTPS06	TCCGTGCCACAGATCCACTACC	TCGGTACAACTTTCCCTCCTTTGC
RcTPS07	CGAATCCACTACGCCAGAGAAGC	TTAGCAGAGGGTAGCCTGTGGTC
RcTPS08	CAGGCTACCCGCTGCTTGTAAC	TGCACACCCTTTCACAGGTTTGG
RcTPS09	AGCATTATTGGTGGACGCAGAGTG	GCCCCTTCATCATGTGTTGTGGAG
RcTPS10	GGTCCTTCACCACCACTCATCTTG	CCTCGCTTCAATCCTTGGGTTCC
RcTPS11	TACCACCACTCATCTCCAGACTGC	CATGTAACGCCGAGCACCTAACC
RcTPS12	CCAGCATTTGGGGCGATCATTTTC	AGCACGCTCTTCACTTCTTCCTTC
RcTPS13	GAGACACCGATTTGAGGCCA	CCCATGCTGTCTAAGGAGCC
RcTPS14	AAGCACTAACCTTCACCACCACAC	TTCTAGCCTCGGTAAGCCCTTCC
RcTPS15	TGGTGGAAGGACTTGGACGTGAG	CACTGACAAAGCCCAGCCGAAG
RcTPS16	GCACTGACCTTCACCACCACAC	TTCTAGCCTCGGTAAGCCCTTCC
RcTPS17	CACTAACCTTCACCACCGCTCATC	CCTCGCTTCAATCCTGGGCATG
RcTPS18	CGAATCCACTACGCCAGAGAAGC	TAGCATAGGGTAGCCTGTGGTCAC
RcTPS19	TGCGACGACAGCTTCAAGGATTG	GCTAGTGAATGCAAGGGCCTCATC
RcTPS20	TGGCGTCACAACAAGTACACACC	ACCAGAATGACCACCCCGGATAC
RcTPS21	ATGTTGTCTCAGCGGTGGAATGC	AGGGACAGTAGCGATAGGGTGAAG
RcTPS22	GCCAGATGGTTCCACGAAGGATG	ATGGTGTTACCGACAGAAGTTGCC
RcTPS23	CACTAACCTTCACCACCGCTCATC	ACAAGTGATGCCTCGCTTCAATCC
RcTPS24	CAACCATGCCTGTCCAAGCTACTC	TGTGCGCCGAACAACTTCTGG
RcTPS25	TTCAAAGACGAGCGAATCCCAACC	CAATATCTCCCATGCCGACCAAGG
RcTPS26	AGCATTTGGGGCGATCAGTTTCTC	CGGAGCCTTCAGCATCGTGTTC
RcTPS27	AGGCACTATCCTTCACCACCACTC	CCACCCTGTTGATGCCCTTTCG
RcTPS28	TCTAATGCGACCTCTCAACCTTGC	TAGGCAGTAGGCACGGGTTCG
RcTPS29	CAAATCAACAGGTCGGGACAGGAG	GGTGGCTGATGGAAACTGGATGAG
RcTPS30	ACCCCAATACTTGCTTGCCAGAAG	AGTTCGTCAATGGTGCCGTATGC
RcTPS31	GAGGCAACAGAAGCAACTCAGAGG	AAGCAGCAGCAGTAGTTGATGGTG
RcTPS32	GGTCCTTCACCACCACTCATCTTG	AATGCCTCGCTTCAATCCTTGGG
RcTPS33	GCATTATTGGTGGAGGCAGAGTGG	GGCATGAGTCAAAGTCAGCAAAGC
RcTPS34	CCCGCCGCTTTTCAAACAAGTAAG	TTGGTTGCGTGATCCGTGTAGTTC
RcTPS35	TCGTCCACAGCAGCAATTCTTCG	TCGTGCCTCGTCAATGGAACAAC
RcTPS36	TCCGTGCCACAGATCCACTACC	TCGGTACAACTTTCCCTCCTTTGC
RcTPS37	TTCCACGGAGAAGAAACGCTTGAC	GATGCTCCAGTGTGTGCGAGAC

RcTPS38	GGTCCTTCACCACCACTCATCTTG	AATGCCTCGCTTCAATCCTTGGG
RcTPS39	GCTTGCGAAGGAGGGGAAGTTG	TTGTGGAAAGAGCGGCTGACATG
RcTPS40	TCGTCCACAGCAGCAATTCTTCG	GCCTCGTCAATGGAACAGCCTTG
RcTPS41	CGACTTTGGGACGACTTGGGAAG	TGCCTCCTCAATGGAACAACCTTG
RcTPS42	AGGACGCACTCCGAGCATGG	TTTCGGGTCATCCAACAACCACTC
RcTPS43	TACCACCACTCATCTCCAGACTGC	CATGTAACGCCGAGCACCTAACC
RcTPS44	AGGCACTAGACTTCACCACCACTC	TCTTGCTTCCACCCTCGGGATG
RcTPS45	GAAGTGAGGAGGACGCTAATGGC	GGACACGCCTAGACGCTGAATG
RcTPS46	TCAACATGGTTCAAGCCACACTCC	CCCACAGCACACATGAAACATTCG
RcTPS47	TGCTCAAGGCTATGCTGAAGGAAG	GGTCCCAAGGCAAATGAGACGTAC
RcTPS48	TACCACGACTCATCTCCAGACTGC	CATGTAACGCCGAGCACCTAACC
RcTPS49	GTTCCTGACGAGTCTCCTGTGTTG	CCAGTCAAGACCTGCCATTCCTTC
RcGAPDH	GCTGGCAGGTATCCTTTCTG	GGCGACAATATCAGCCAAGT

Table S6. Functional annotation of RcTPS proteins by Terzyme (http://www.nipgr.ac.in/terzyme.html).

Gene ID	Score	E-value	No. of domains	Putative function
RcTPS01	830.7	1.40E-252	1	Sesquiterpene Synthase
RcTPS02	806.8	2.50E-245	1	Sesquiterpene Synthase
RcTPS03	805.4	6.30E-245	1	Sesquiterpene Synthase
RcTPS04	800	2.80E-243	1	Sesquiterpene Synthase
RcTPS05	796.4	3.40E-242	1	Sesquiterpene Synthase
RcTPS06	792.3	5.80E-241	1	Sesquiterpene Synthase
RcTPS07	792.2	6.20E-241	1	Sesquiterpene Synthase
RcTPS08	792.1	6.80E-241	1	Sesquiterpene Synthase
RcTPS09	784.3	1.50E-238	1	Sesquiterpene Synthase
RcTPS10	783.9	2.10E-238	1	Sesquiterpene Synthase
RcTPS11	780.1	2.90E-237	1	Sesquiterpene Synthase
RcTPS12	779.6	4.30E-237	1	Sesquiterpene Synthase
RcTPS13	776.9	2.80E-236	1	Sesquiterpene Synthase
RcTPS14	776.6	3.40E-236	1	Sesquiterpene Synthase
RcTPS15	771.1	1.60E-234	1	Sesquiterpene Synthase
RcTPS16	770.3	2.80E-234	1	Sesquiterpene Synthase
RcTPS17	768.8	7.70E-234	1	Sesquiterpene Synthase
RcTPS18	767.3	2.30E-233	1	Sesquiterpene Synthase
RcTPS19	767.3	2.30E-233	1	Sesquiterpene Synthase
RcTPS20	763.8	2.60E-232	1	Sesquiterpene Synthase
RcTPS21	761.7	1.10E-231	1	Sesquiterpene Synthase
RcTPS22	761	1.90E-231	1	Sesquiterpene Synthase
RcTPS23	760.3	2.90E-231	1	Sesquiterpene Synthase
RcTPS24	755.5	8.20E-230	1	Sesquiterpene Synthase
RcTPS25	751	1.90E-228	1	Sesquiterpene Synthase
RcTPS26	750.8	2.20E-228	1	Sesquiterpene Synthase
RcTPS27	733.9	2.90E-223	1	Sesquiterpene Synthase
RcTPS28	720.7	2.90E-219	1	Sesquiterpene Synthase
RcTPS29	715.8	9.00E-218	1	Sesquiterpene Synthase
RcTPS30	706.3	6.50E-215	1	Sesquiterpene Synthase
RcTPS31	705.6	1.10E-214	1	Sesquiterpene Synthase
RcTPS32	683.7	4.70E-208	2	Sesquiterpene Synthase
RcTPS33	623.6	7.30E-190	2	Sesquiterpene Synthase
RcTPS34	611	5.00E-186	1	Sesquiterpene Synthase
RcTPS35	692.8	8.40E-211	1	Monoterpene Synthase
RcTPS36	672.6	1.10E-204	1	Monoterpene Synthase
RcTPS37	664.9	2.50E-202	1	Monoterpene Synthase

RcTPS38	622.6	1.60E-189	1	Monoterpene Synthase
RcTPS39	622.1	2.20E-189	1	Monoterpene Synthase
RcTPS40	616.3	1.20E-187	1	Monoterpene Synthase
RcTPS41	555	4.70E-169	1	Monoterpene Synthase
RcTPS42	552.2	3.20E-168	1	Monoterpene Synthase
RcTPS43	542.4	3.00E-165	1	Monoterpene Synthase
RcTPS44	503.5	1.80E-153	1	Monoterpene Synthase
RcTPS45	956.4	2.90E-290	1	Diterpene Synthase
RcTPS46	848.4	1.30E-257	2	Diterpene Synthase
RcTPS47	822.1	1.10E-249	2	Diterpene Synthase
RcTPS48	688.9	2.00E-209	1	Diterpene Synthase
RcTPS49	575.7	3.60E-175	1	Diterpene Synthase
